# The Diverse Roles of Mitochondria in Regulating Cancer Metastasis

**DOI:** 10.3390/cimb47090760

**Published:** 2025-09-15

**Authors:** Shiyu Tang, Biao Yang

**Affiliations:** 1 Department of Pathophysiology, Shenyang Medical College, Shenyang. No. 146, Huanghe North Street, Shenyang 110034, China; wnzgstsy@symc.edu.cn; 2 Department of Pathogen Biology, Shenyang Medical College, Shenyang. No. 146, Huanghe North Street, Shenyang 110034, China

**Keywords:** mitochondrial, metastasis, dissemination, dormancy, colonization, microenvironment

## Abstract

Metastasis is the primary cause of cancer-related deaths. As a multi-step process, tumor metastasis encompasses several key aspects. Tumor cells first traverse the basement membrane and subsequently invade the surrounding vascular or lymphatic systems, ultimately leading to secondary colonization. Throughout the progression of metastasis, tumor cells can overcome selective pressures and transition between different cellular states, depending on the diverse functions of mitochondria. Mitochondria not only function as energy generators but also co-evolve with host cells, acting as critical signaling hubs in various biological pathways. Under sustained stress conditions such as nutrient deficiency, cellular stress, and the reprogramming of gene expression, alterations in mitochondrial morphology and function can prevent cell death and facilitate the targeted transformation of oncogenes, tumor progression, and the emergence of invasive cell phenotypes. The multifaceted roles of mitochondria enable tumor cells to evade unfavorable environments and establish colonies in more conducive sites. In summary, this review consolidates the complex interactions between mitochondria and cancer while elucidating their significant role in cancer metastasis and therapeutic responses.

## 1. Introduction

Metastasis, characterized as a continuously evolving and heterogeneous systemic disease, remains the primary cause of cancer-related mortality [1]. Nevertheless, our comprehension of the mechanisms by which cancer cells escape from the primary tumor site to distant organs—and how to effectively treat this phenomenon—is still developing. Cancer cells undergo clonal selection and phenotypic alterations while simultaneously influencing their immune microenvironment [2]. These malignant cell seeds can infiltrate various organs via blood circulation, lymphatic vessels, or direct invasion through adjacent tissues [3]. At the metastatic site, if these seeds successfully adapt to the local microenvironment and navigate numerous obstacles, they may ultimately resume proliferation in nearby or distant organs and establish successful colonization [4].

According to the Warburg effect hypothesis, damage to the respiratory chain coupled with enhanced aerobic glycolysis are considered pathogenic factors in tumor development [5]. Glycolysis and mitochondrial adenosine triphosphate (ATP) synthesis represent two principal pathways for ATP production; glycolysis is optimized for rapid ATP generation while mitochondrial processes are tailored for maximal ATP yield [6]. The emergence of tumors necessitates both glycolysis and oxidative phosphorylation (OXPHOS) for energy production and large-molecule synthesis [7]. Therefore, a comprehensive understanding of the roles played by glycolysis and OXPHOS in tumor growth and progression is essential. Similarly, metastasis requires meticulous regulation of mitochondrial function to withstand ongoing selective pressures [8]. Analyzing heterogeneity among tumor cells across primary, invasive, and metastatic stages holds significant implications for preventing and treating metastatic cancers.

Metastasis typically encompasses several fundamental stages, including initial initiation and dissemination, long-distance transport and dormancy, as well as colonization of various organs [9]. The tumor microenvironment (TME) and prolonged drug therapy also significantly influence the metastatic process. Mitochondria, along with other organelles and cellular structural components, can effectively regulate and adapt to metastatic stress while mitigating stress-induced damage. This adaptability contributes to alterations in cancer cell survival, migration, and plasticity [10]. In summary, our review elucidates the intricate relationship between mitochondria and cancer, highlighting their critical roles in metastasis and therapeutic responses.

## 2. Search Strategy and Selection Criteria

A non-systematic literature review was conducted through a comprehensive literature review of publications indexed in the PubMed medical database from 1 January 2015 to 1 June 2025. Additionally, we incorporated relevant studies that addressed particular issues encountered during the writing process. The following keywords were utilized: “Mitochondrial”, “Metastasis”, “Dissemination”, “Dormancy”, “Colonization”, and “Microenvironment”. This review included review articles and clinical guidelines that examined the progression of metastasis across the dynamic changes in mitochondria from morphology to function. Only peer-reviewed, full-text articles published in English within the past ten years were selected. Particular emphasis was placed on “Basic Research”, “Clinical Study”, and “Review” article types that were directly relevant to the subject matter. The exclusion criteria encompassed duplicate publications, studies lacking diagnostic outcomes, case reports, correspondences, letters to the editor, and research involving non-human subjects.

## 3. Dissemination

After tumor resection surgery, patients may experience a period of several years or even longer without significant issues; however, they ultimately face relapse and are diagnosed with advanced cancer, which presents a perplexing outcome [11]. This phenomenon is most likely attributable to the dissemination of residual tumor cells or the emergence of micrometastases that remain undetectable through clinical methods [12]. Furthermore, it may also be due to early disseminated tumor cells entering a dormant state and acquiring resistance until they are unexpectedly activated, leading to proliferation and the formation of more pronounced metastatic lesions [13]. Metastasis is a multifaceted process that involves intricate interactions between tumor cells and the host microenvironment, which encompasses various components, such as the extracellular matrix, immune cells, and vascular endothelial cells. There are three primary pathways through which tumor metastasis occurs: firstly, hematogenous metastasis, in which tumor cells disseminate to distant organs via the bloodstream; secondly, lymphatic metastasis, where tumor cells propagate through the lymphatic system; and then, implantation metastasis, characterized by the direct invasion of adjacent tissues or organs by tumor cells. The propensity for metastasis varies among different tumors based on their biological characteristics and anatomical locations. For example, intestinal tumors typically metastasize to the liver as an initial site, while lung cancer is particularly susceptible to metastasizing to the brain.

### 3.1. Stress Selection

On the initial stage of metastasis, a subset of cancer cells is subjected to heightened migratory and invasive stimulation signals. Numerous studies have indicated that factors such as hypoxia, nutrient deficiency, and oxidative stress can impose selective stress on cancer cells, thereby affecting mitochondrial function and promoting migration and invasion [14,15]. Cancer cells, in order to survive, must adapt to the stress-induced injuries produced predominantly by mitochondrial regulation. A detailed understanding of the mitochondrial stress response and associated cellular pathways will be essential for cell survival mechanisms and may provide potential therapeutic targets for monitoring cancer progression [16].

**Figure 1:**
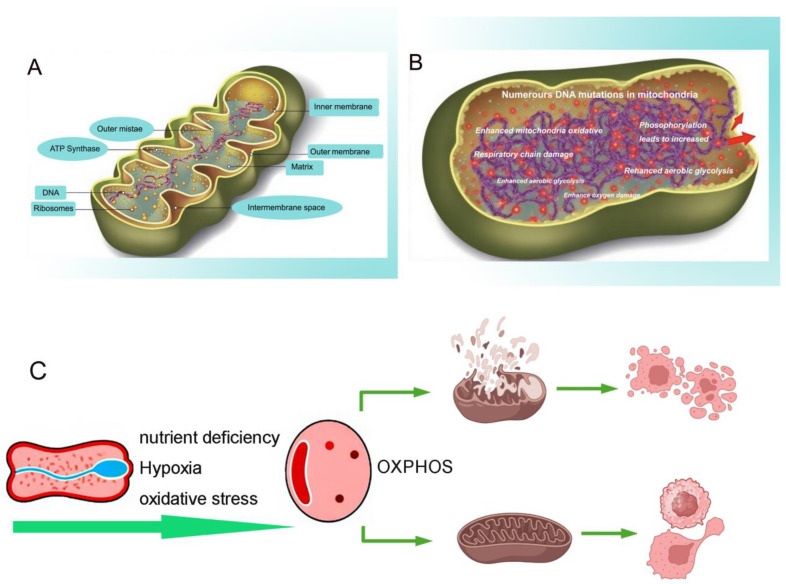
The alterations in mitochondrial morphology and functionality observed in subtype cells under stress selection. (**A**) The the structure and morphology of mitochondria under normal physiological conditions. (**B**) The morphology and function of mitochondria under pathological stress conditions. Numerous DNA mutations can be observed in mitochondria; enhanced mitochondrial oxidative phosphorylation leads to increased respiratory chain damage, a decrease in aerobic glycolysis; enhanced oxygen damage, and enhanced aerobic glycolysis. (**C**) On the left, the irregular elongated strip/net-like structure represents the ER, while the oval structure denotes the mitochondria. The intervening region between these two organelles indicates the MAM contact site, which facilitates calcium exchange and functional interactions. The central figure illustrates that mitochondria house both the TCA cycle and the OXPHOS, with small particles representing mtDNA. The mitochondrial diagram located in the upper right corner illustrates the OMM localization of pro-apoptotic proteins Bak and Bax, which induces mitochondrial permeability and facilitates cytochrome c release. They trigger apoptosome formation and activate caspase proteases in the cytosol, ultimately leading to cell death. The mitochondrial diagram located in the lower right corner illustrates cancer cells that can elevate the threshold for mitochondrial apoptosis by activating specific mitochondrial biological processes. These cancer cells frequently induce mitochondrial biogenesis and undergo metabolic reprogramming to meet energy demands while producing essential biomolecules for sustained cell division and oncogenic signaling. The image was created using Biorender [26].

Mitochondria are organelles that are encased by two distinct membranes. The outer membrane is smooth, whereas the inner membrane exhibits inward folds known as cristae. The region between the inner and outer membranes is referred to as the intermembrane space, which contains a matrix. The mitochondrial matrix houses all the enzymes necessary for the tricarboxylic acid cycle (TAC) and provides biosynthetic intermediates essential for anabolic processes involving lipids, sugars, proteins, amino acids, and nucleotides. Additionally, it serves as the site where sugars, fats, and amino acids undergo oxidation to release energy. Mitochondria not only function as metabolic powerhouses but also co-evolve with the host, playing a crucial role in various signaling pathways [17]. The mitochondrial enhances the efficiency of electron transfer through the formation of supercomplexes. Additionally, it minimizes the generation of reactive oxygen species and safeguards cells against oxidative damage. Figure 1 shows the alterations in mitochondrial morphology and functionality observed in subtype cells selected under stress selection. An increasing number of studies have demonstrated that stress conditions such as hypoxia, metabolic stress, and proliferative stress can activate reactive oxygen species (ROS), which are primarily generated through mitochondrial OXPHOS [18,19]. This activation leads to a mutation rate of mitochondrial DNA (mtDNA) that is more than ten times higher than that of nuclear DNA (nDNA), resulting in impaired mitochondrial function in cancer cells [20]. Consequently, this phenomenon is becoming a driving factor for malignant tumors.

To enhance their survival and proliferative capabilities, cancer cells engage various stress response pathways to counteract both exogenous and endogenous stressors [21]. To mitigate harmful effects, the active or passive metastasis of cancer cells necessitates the involvement of mitochondria [22]. Mitochondrial biogenesis is regulated by nDNA as well as mtDNA. Mutations in either nDNA or mtDNA can compromise mitochondrial function and induce mitochondrial stress, resulting in dysregulation of cellular signaling pathways [23]. Mitochondria have evolved specialized pathways to alleviate various stresses, including the unfolded protein response (UPR), mitochondrial fission and fusion processes, and mitophagy [24]. Research has demonstrated that mTOR promotes mitochondrial biogenesis while inhibiting autophagic elimination of mitochondria [25].

The interaction between the endoplasmic reticulum (ER) and mitochondria plays a critical role in determining the replication, division, and distribution of mitochondria [27]. This contact regulates both mitochondrial division and mtDNA synthesis. Additionally, the distribution of mitochondria is influenced by their processes of fission, fusion, and movement [28]. To ensure accurate mtDNA distribution within cells, it is essential that mitochondrial fission associated with the ER occurs effectively. The positioning of mtDNA during division dictates the site of contact between the ER and the outer membrane of mitochondria. Within cells, there are numerous such contact points that influence where mtDNA division takes place and how mitochondria undergo division; however, mtDNA division occurs at only a small subset of these contact sites [29].

The ER is a crucial organelle within cells, primarily responsible for protein processing and transportation. Additionally, it plays a significant role in regulating the balance of intracellular calcium ion concentrations [30]. The exchange of information between the ER and mitochondria can profoundly influence cellular fate. Mitochondria coordinate various cellular activities by modulating calcium ion concentrations [31]. During instances of ER stress, an excessive influx of calcium ions may occur uncontrollably along the mitochondria-associated ER membrane (MAM), transferring from the ER to the mitochondria. This results in an overload of calcium ions within the mitochondria, which accelerates cell apoptosis [32]. Conversely, in the outer mitochondrial membrane (OMM), the localization of pro-apoptotic proteins Bak and Bax induces mitochondrial permeability and facilitates cytochrome c release. This release triggers apoptosome formation and activates caspase proteases in the cytosol, ultimately leading to cell death [33]. Furthermore, BCL-2 and other members of anti-apoptotic protein families effectively inhibit tumor cell death/apoptosis by preventing excessive accumulation of Bax on the OMM [34,35].

Cancer cells depend on the mitochondrial OXPHOS system to sustain their high proliferation capacity [36]. The TCA cycle and fatty acid oxidation (FAO) occur within the mitochondrial matrix. Intermediates generated from TCA metabolism serve as substrates for amino acid biosynthesis and maintaining redox balance, while FAO provides an alternative energy source for ATP production [37]. In addition to ATP, mitochondria synthesize various macromolecules that are crucial for cellular function [38]. Importantly, mitochondria are involved in the synthesis of amino acids, fatty acids, cholesterol, and numerous metabolic intermediates that can function as signaling molecules. Within mitochondria, the synthesis of fatty acids and cholesterol can be upregulated as components of the lipid bilayer membrane—a fundamental requirement for cell division [39]. Consequently, cancer cells frequently induce mitochondrial biogenesis and undergo metabolic reprogramming to meet energy demands while producing essential biomolecules necessary for sustained cell division and oncogenic signaling [40]. A hallmark characteristic of cancer is its ability to evade apoptosis; these cells can elevate the threshold for mitochondrial apoptosis by activating specific mitochondrial biological processes [41]. In cellular physiology, mitochondria play a pivotal role in influencing cell viability and functionality.

In the face of the stress selection, the glucose levels of tumor cells are crucial for physiological function. The core regulator of metabolism, AMP-activated protein kinase (AMPK), is further activated by decreased glucose levels [42]. In conditions of glucose starvation, without an increase in AMP levels, the lysosomal pool of AMPK is exclusively activated. According to changes in AMP levels, cells activate differentially compartmentalized pools of AMPK [43,44]. When glucose levels decline, AMPK and LKB1 bind to the ATPase complex region and regulatory sites on the lysosomal membrane via the AXIN protein. Here, AMPK is activated by LKB1, initiating downstream catabolic pathways [45]. Varying degrees of energy deficiency can progressively activate AMPK in distinct cellular regions.

In primary markers of metastasis—gene expression regulation, metabolic adaptation, and phenotypic plasticity—cells exhibit a dynamic ability to non-genetically adapt to diverse pressures [46,47]. These studies underscore the significance of cell plasticity in cancer progression, wherein identical molecules and pathways assume different roles at various stages during proliferation and metastasis.

### 3.2. Transdifferentiation

Metastatic cells represent a subset of cells capable of adapting to their surrounding environment. These cells exhibit phenotypic characteristics akin to those of primary tumors and have been identified as genetically heterogeneous within the primary tumor [48]. This adaptive selection process enables tumors to respond more effectively to various dynamic changes in tissues, including the loss of cell polarity and downregulation of epithelial cell adhesion molecules, thereby enhancing their ability to migrate and invade adjacent tissues.

Epithelial–mesenchymal transition (EMT), which is essential for tumor progression, serves as a prime example of plasticity program selection [49]. During EMT, cells lose their epithelial identity and acquire mesenchymal traits—processes that are not only critical during development and wound healing but also constitute significant features in both primary tumor formation and metastasis. EMT plays a pivotal role in augmenting metastatic potential. Core evidence supporting the occurrence of EMT is derived from studies manipulating EMT transcription factors (EMT-TFs), indicating that EMT transpires during the early stages of primary tumor progression, with distinct EMT states coexisting throughout the metastatic process [50].

This program encompasses multiple dynamic alterations in cellular organization, including the loss of cell polarity and downregulation of epithelial cell adhesion molecules. These changes culminate in an enhanced capacity for migration and invasion into adjacent tissues [51]. This process is driven by the coordinated and dynamically regulated functions of Snai1, Snai2, Twist1, Zeb1, and Zeb2—transcriptional repressors that target epithelial genes [52]. The EMT transcription factors capable of inducing the classic EMT program are often co-expressed.

Nuclear genes regulate mitochondrial biogenesis to synthesize additional mitochondria, thereby fulfilling various requirements for cellular survival. Mitophagy represents a specialized form of autophagy that selectively degrades and eliminates excess or damaged mitochondria [53]. The synergistic interplay between mitochondrial biosynthesis and autophagy governs both the quality and functionality of mitochondria. Conversely, defective mitochondria can directly emit signals that trigger apoptosis. The mitophagy mechanism involves membrane depolarization as well as cascades of mitochondrial protein phosphorylation and ubiquitination; meanwhile, diminished cellular energy output activates mitochondrial biogenesis through universal energy sensors such as AMP kinase, among other pathways [54].

AMPK is a serine/threonine protein kinase that functions as an energy sensor, regulating various physiological systems, including glucose and lipid metabolism. At elevated O_2_ concentrations (1–3%), nitric oxide (NO) promotes the production of reactive oxygen species, which in turn activates AMPK through a mechanism that is independent of nucleotide concentrations [55]. AMPK enhances skeletal muscle mass by inhibiting the activity of mechanistic target of rapamycin complex 1 (mTORC1) and promoting protein synthesis via the regulation of ubiquitin–proteasome pathways and autophagy for degradation processes [56]. Furthermore, AMPK activation increases the transcriptional activity of peroxisome proliferator-activated receptors and nuclear respiratory factors (NRFs) through phosphorylation of PPAR-γ coactivator 1 (PGC-1). PGC-1 serves as a master regulator involved in mitochondrial respiration and biogenesis. It interacts with NRFs at the promoter region of mitochondrial transcription factor A (mtTFA) to facilitate their activation [57].

Recent research has demonstrated that AMPK plays a critical role in modulating signaling pathways associated with skeletal muscle hypertrophy and atrophy [58]. These morphological changes indicate that the AMPK pathway is integral to regulating plasticity adaptations within skeletal muscle.

Plasticity refers to the ability of cells to adapt to constantly changing environments. Once cancer cells invade the tumor stroma, they possess the ability to disseminate into circulation and colonize distant organs [59]. Tumor cells in a complete EMT state invade surrounding tissues as mesenchymal single cells, whereas hybrid EMT states facilitate migration; tumor cells at the leading edge exhibit a more pronounced EMT phenotype [60]. The migration of hybrid EMT cells is associated with plasticity, desiccation, invasiveness, and an enhanced metastatic potential.

### 3.3. Migration out of Nest

During the dissemination process, tumor cells influenced by TME factors that promote oncogenic mutations or those selected for their own heterogeneity can invade deeper tissue layers and acquire survival capabilities [61]. Subsequently, these cells infiltrate proximal blood vessels or lymphatic vessels, ultimately spreading to adjacent spaces such as the peritoneum or pleural cavity through mechanisms including endothelial migration, capillary rupture, or direct local diffusion. This allows them to extravasate into distant organs. It is important to acknowledge that escaping from the primary site represents a significant bottleneck in metastasis. During cancer cell invasion, more rapidly migrating cancer cells play a pivotal role in facilitating further invasion and earlier metastatic events [62].

Beginning with the Warburg effect, tumor cells shift their metabolic preference from mitochondrial OXPHOS to aerobic glycolysis for energy production [63]. Nevertheless, mitochondria and OXPHOS continue to be crucial in tumor metastasis and dissemination. Research indicates that regulating mitochondrial shape, size, morphology, and metabolism is essential for sustaining localized high-energy environments [64]. In the metastatic phase of tumor progression, energetically active mitochondria relocate to the surface cytoskeleton of tumor cells; this relocation is also a prerequisite for enabling these cells to invade the basement membrane and successfully metastasize.

**Figure 2:**
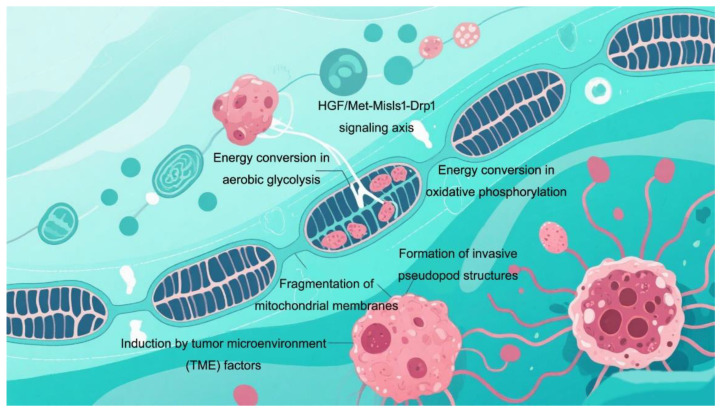
Illustration of tumor cell progression involving mitochondrial mechanisms, depicting the signaling axis formation of HGF/Met-Misls1-Drp1, energy conversion processes (aerobic glycolysis and oxidative phosphorylation), mitochondrial membrane fragmentation, formation of invasive pseudopodia, and the induction role of TME factors in tumor cell invasion and metastasis.

The efficiency of mitochondrial translation is essential for maintaining cytoplasmic protein homeostasis and regulating nuclear stress signaling, thereby directly influencing cellular lifespan. The HGF/Met-Mis1-Drp1 axis plays a significant role in mitochondrial fission and metastasis [65]. Following fission, fragmented mitochondria that localize to the leading edge of the cell can supply the energy necessary for actin rearrangement and pseudopodia formation, including lamellipodia and invadopodia, thus facilitating metastatic processes [66]. Metastasis-initiating cells (MICs) require elevated mitochondrial activity to support invasion [67]. Cell movement and invasion rely on the localized generation of ATP within mitochondria to coordinate the reorganization of focal adhesions (FAs) and the actin cytoskeleton [68]. Actin aggregation and new focal adhesion formation occur at the anterior edge in alignment with directional movement, while actin contraction and focal adhesion disassembly are confined to the posterior edge. Mitochondrial rhoGTPases, such as miro-1 and miro-2, are crucial for distributing mitochondria along microtubules [69]. Furthermore, disrupting mitochondrial redistribution through fusion diminishes the number of mitochondria positioned at the front end of cells. This impairment hinders their ability to migrate rapidly and directionally within constrained environments (Figure 2).

The mechanical regulation of cytoskeleton remodeling during diffusion and migration entails a metabolic shift towards enhanced OXPHOS, which is essential for membrane wrinkling [70]. Research has demonstrated that the localized accumulation of mitochondria in the limbic peduncle holds bioenergetic significance in cancer cell migration through mechanisms such as membrane protrusion and focal adhesion stability. The reverse metabolic switch from glycolysis to OXPHOS facilitates tumor cell invasion and metastasis. Mitochondrial RNA plays a crucial role in regulating the metabolic reprogramming necessary for invasion and dissemination [71]. Invading leader cells exhibit an upregulation of mitochondrial membrane potential (MMP) alongside increased mitochondrial lengths [72]. Furthermore, modifications to mitochondrial tRNA regulate the rate of mitochondrial translation, thereby driving the metabolic reprogramming required for metastasis. The upregulation of CD36 enhances fatty acid uptake for lipid homeostasis but can also support mitochondrial respiration under stress conditions [73]. Additionally, researchers have identified that CD36-dependent non-dividing metastatic tumor cells necessitate mitochondrial m5C modification to activate invasion and dissemination processes [74]. Notably, CD36 expression correlates with poor patient survival across various cancer types.

OXPHOS remains a critical driving factor for tumor growth, facilitating essential characteristics such as cellular movement and metastasis. However, it is associated with abnormal production of ROS, increased fatty acid metabolism, and remodeling of OXPHOS. In response to stressors within the local tumor microenvironment, mitochondria act as partial providers of bioenergy; their dynamic distribution among organelles determines their localization as membrane protrusions that facilitate cell movement, thereby promoting chemotaxis and enhancing cell invasion [75]. Research has demonstrated that the SNPH pathway reprograms mitochondrial dynamics to support enhanced cell motility and enable escape from unfavorable ecosystems [76]. SNPH regulates the speed of mitochondrial transport, preventing inactivated or damaged mitochondria from relocating to the cortical cytoskeleton, thus ensuring optimal survival efficiency for tumor cells.

In the disseminated tumor cell cycle, circulating tumor cells (CTCs) experience extensive damage due to physical stressors, redox imbalances, and immune challenges. Conversely, mitochondria empower cells to rapidly adapt to environmental changes, thereby increasing their chances of survival [77]. CTCs circulate either as single cells or stem-like cancer cells surrounded by platelets, neutrophils, or tumor-derived stromal cells. This protective environment shields them from immune surveillance while endowing CTC clusters with greater metastatic potential compared to individual cells [78]. Consequently, they play a pivotal role in various aspects of tumor development.

## 4. Dormancy

During the initial stages of metastasis, local invasion occurs alongside the establishment of distant metastatic sites and colonization, as well as evasion of the immune system. In the dispersal phase, the microenvironment can induce a regulatory program that leads to reversible growth arrest in transferred cells, causing them to enter a quiescent state [79]. This state of cellular plasticity is referred to as dormancy, wherein cells become truly dormant. Consequently, these dormant cells can evade conventional detection methods. If this incubation period is successful, they will typically only be identified through clinical examination following recurrence.

### 4.1. Dedifferentiation

The metabolic adaptation of the TME further exacerbates the slow cell cycle state induced by tumor hypoxia or nutrient deprivation. Indeed, compelling evidence now demonstrates that cancer heterogeneity not only arises from genetically distinct subclones but is also driven by phenotypic and functional diversity [80].

Mitochondria generate ATP through OXPHOS, thereby influencing cell fate via energy conversion regulation. In differentiated cells, mitochondrial damage disrupts energy supply and leads to the accumulation of ROS [81]. This disruption can result in cellular structural damage through the activation of ferroptosis. Mitochondrial ROS play a regulatory role in pluripotent stem cells; it is generally accepted that low levels of ROS help maintain genomic integrity, while elevated levels may promote differentiation.

Under stress conditions, cells employ mitophagy to eliminate dysfunctional or excessive mitochondria. The upstream signals regulating mitophagy include mitochondrial fission, AMPK, activating transcription factor 4 (ATF4), and mtDNA release, among others [82]. Mitochondrial stress responses are triggered by respiratory chain inhibition and mutations within the mitochondrial genome. The aggregation of mitochondrially localized proteins in the cytoplasm creates an imbalance between mitochondrial and nuclear-encoded proteins; antibiotics can induce defects in mitochondrial translation as well as mitophagy [83]. Furthermore, Pink1/Parkin deficiency has been shown to regulate HIF1α expression, which influences glycolysis, angiogenesis, and metastasis [84].

The interaction between ER proteins and microtubules plays a crucial role in regulating the movement of organelles along differentially modified microtubules, thereby establishing and maintaining their appropriate distribution and function [85]. The ER extends as a network of various forms within the cytoplasm, forming connections with other organelles. Three types of membrane-bound ER proteins exhibit preferential interactions with distinct microtubules: cytoskeleton associated protein 4 (CKAP4) binds to centrosomal microtubules, kinin associates with perinuclear polyglutamylated microtubules, and P180 interacts with glutamylated microtubules [86]. The knockout of these proteins or manipulation of the states of microtubules and glutaraldehyde can lead to significant alterations in the positioning of the ER, resulting in similar redistributions of other organelles. Under conditions of nutrient deficiency, cells modulate CKAP4 protein levels and P180 binding to microtubules to facilitate bidirectional movement between the endoplasmic reticulum and lysosomes, thus achieving appropriate autophagic responses.

The Regulated in Development and DNA Damage Response-1 (REDD1) protein acts as an effector for hypoxia-inducible factor 1 (HIF-1), and it serves as an essential regulator of TORC1 activity [87]. Conversely, loss of REDD1 leads to stabilization of HIF-1 through a ROS-dependent mechanism. In REDD1 knockout cells, a hypoxic environment can inhibit TORC1 expression. Consequently, antioxidant treatment is sufficient to normalize HIF-1 levels while inhibiting REDD1-dependent tumor formation. Furthermore, REDD1 functions as a direct regulator of mitochondrial metabolism; it localizes to mitochondria—a localization that is necessary for reducing ROS production [88].

In research on paligenosis, it has been observed that when cells are exposed to unfavorable environments or subjected to injury stimuli, the primary energy regulatory target TORC1 is downregulated. This leads to an increase in autophagy lysosomal activity, which facilitates the degradation of cellular subunits, while simultaneously enhancing the expression of regulatory progenitor and stem cell genes. Subsequently, TORC1 is re-expressed, promoting re-entry into the cell cycle. During this process, several key genes associated with monitoring DNA damage—namely interferon-related developmental regulator 1 (IFRD1), REDD1, and the p53 tumor suppressor gene (TRP53)—play significant regulatory roles [89]. Furthermore, the transcriptional axis involving mitochondrial co-transcriptional activator PGC-1 and its downstream target transcription factor NRF2 indicates a transcriptional program that mitigates excessive ROS effects [90].

### 4.2. Restore Stem Cell Characteristics

Cellular plasticity is a key characteristic of dormancy. Recent evidence highlights that disseminated tumor cells (DTCs) activate early mesenchymal-like programs associated with pluripotency plasticity, which coordinate dissemination and achieve long-term dormancy controlled by the transcription factor ZFP281 [91]. Research data indicate that in the hypoxic TME, high expression of Glutaminase 1 (GLS1) promotes carcinoma metastasis, potentially involving the induction of cancer stem cell (CSC) phenotypes [92]. Thus, DTCs serve as seeds that support specific organ niches with fertile nutrients to facilitate metastasis. Notably, four transcription factors—organic cation/carnitine transporter4 (OCT4), SRY-box transcription factor 2 (SOX2), KLF transcription factor 4 (KLF4), and myelocytomatosis oncogene (c-MYC)—are sufficient to reprogram differentiated somatic cells into induced pluripotent stem cells (iPSCs) [93]. Stem cell pools can proliferate necessary stem cells to adapt to their environment.

Mitochondria have been reported to play essential roles in determining cell fate. The regulation of mitochondrial signaling can reprogram somatic cells into iPSCs and modify chromatin configurations. It has been shown that the mitochondrial permeability transition pore (mPTP) is a key regulator of mitochondrial homeostasis; it mediates epigenetic regulation representing a novel mitochondria-to-nucleus pathway in cell fate determination [94].

Cancer stem cells are dependent on OXPHOS with very limited metabolic plasticity, while non-CSCs exhibit high glycolytic activity. Mechanistically, downregulation of MYC followed by an increase in PGC-1α has been identified as a critical determinant for OXPHOS activation [95]. Additionally, low MYC expression levels in human CSCs allow for elevated PGC1A expression levels, resulting in enhanced mitochondrial biogenesis, increased mitochondrial activity and antioxidant properties, ultimately leading to reduced mitochondrial reactive oxygen species levels—a prerequisite for maintaining their stemness functions.

## 5. Colonization

The primary objective of metastasis is to ensure continuous survival. Cancer cells that have entered a state of dormancy must reactivate and re-enter the cell cycle, subsequently proliferating rapidly within the host’s local environment. This local milieu can stimulate the reactivation and growth of DTCs. Ultimately, these DTCs give rise to specific cancers that develop in tissues and organs analogous to those of the primary tumor. These findings underscore the significance of cellular dormancy and subsequent re-differentiation as critical biological processes during metastasis [96].

### 5.1. Differentiation

Mesenchymal–epithelial transition (MET) represents a reversal of EMT, frequently occurring during developmental processes. MET plays a crucial role in adapting to organ-specific microenvironments, inducing pluripotent stem cell reprogramming, restoring the epithelial phenotype, and facilitating metastasis [97]. The deficiency of E-cadherin is associated with increased invasiveness; however, its expression confers protection against oxidative stress while also promoting metastasis and colonization. Tumor cells can utilize E-cadherin to establish specific junctions with N-cadherin expressed by stromal cells within metastatic lesions, thereby enhancing their survival and growth. Notably, most metastatic tumors do not exhibit EMT characteristics, indicating that MET may be critical for successful colonization.

MET plays an essential role in metastatic outgrowth and has significant potential clinical implications that warrant further evaluation regarding therapeutic strategies [98,99]. Twist1 and Prrx1-mediated EMT in carcinoma enhances invasion and circulation of CTCs; conversely, downregulation of these factors promotes metastatic colonization [100]. It is now evident that effective metastatic dissemination necessitates a transition from EMT to MET. Numerous studies suggest that Lgr5+ stem cell populations and proliferation programs are vital for metastatic outgrowth, underscoring the importance of cellular plasticity in the context of metastasis [101]. Furthermore, the organ tropism exhibited by metastatic cells is partially influenced by their metabolic requirements as well as the availability of nutrients within secondary organs.

Importantly, the settlement and colonization of CTCs in distant metastatic target sites critically depend on the metastatic niche within the local inflammatory microenvironment. Infections, tissue injuries, and cellular damage can trigger inflammation, which serves as a key driver of the immune response. Mitochondrial damage-associated molecular patterns released following surgical resection contribute to the formation of pre-metastatic niches in the lungs through IL-1β secretion [102]. At the apex of this hierarchy are populations of CSCs capable of self-renewal, exhibiting long-term in vivo tumorigenicity while generating more differentiated progeny that respond to environmental stress.

The distinctive microenvironment at metastatic sites may influence the reactivation of cells that reach these organs. Tumor cells invading secondary locations have undergone selection for metastasis; theoretically, they exhibit greater invasiveness than primary tumors. Following Drp1 aggregation at mitochondrial–endoplasmic reticulum contact sites—where phosphatidylinositol 4-phosphate [PI(4)P] microregions on trans-Golgi network (TGN) vesicles are recruited—mitochondrial division may be initiated. Furthermore, knockout of ADP ribosylation factor 1 (Arf1) or its effector phosphatidylinositol 4-kinase IIIb [PI(4)KIIIb] can inhibit PI(4)P production and lead to excessive mitochondrial fusion [103]. Postmitotic cells destined for self-renewal undergo mitochondrial fusion; conversely, those maintaining high levels of mitochondrial fission differentiate into neurons [104]. Metastatic cells preferentially migrate to establish a metastatic niche within specific organs. Surviving DTCs may enter a variable period of dormancy during which they either exit the cell cycle or achieve a dynamic balance characterized by bursts of proliferation.

### 5.2. Adhesion and Proliferation

Cancer cells undergo undetectable processes of dispersal and dormancy, allowing them to reach distant organs. Upon colonization in new environments, they establish novel cell–matrix interactions, reshape the ECM, and form micro-transfers. DTCs have the potential to spread to nearly all organs. However, with respect to tumor recurrence, different cancer types exhibit a predilection for specific organs. For instance, following surgical intervention, over 50% of patients with advanced gastric cancer metastasize via the lymphatic system and subsequently spread to the peritoneum [105]. In colorectal cancer, the liver serves as the primary site of metastasis; this occurs as cancer cells disseminate from mesenteric capillaries into the hepatic portal vein before encountering hepatic sinuses [106].

The vast majority of cancer cells that detach from the primary tumor are unable to survive and establish distant metastases. Plasticity is typically necessary to ensure that, following the initial activation of invasive and migratory genes, proliferative genes are re-expressed upon reaching the metastatic site. Under metabolic stress, the dysregulation of the actin cytoskeleton has been extensively investigated in relation to metastatic dissemination [107]. Fascin is directly recruited to mitochondria, where it stabilizes mitochondrial actin filaments (mtF-actin). As an actin-bundling protein, fascin enhances cancer metastatic colonization by increasing resistance to metabolic stress and promoting OXPHOS [108]. The data also clearly indicate a role for both actin and microtubule filaments in tethering and stabilizing dendritic mitochondrial compartments. Single or multiple mitochondrial filaments are spatially stabilized at dendritic regions for extended periods through cytoskeletal anchoring, presumably to support protein synthesis and other energy-demanding processes [109,110]. It is precisely through these intricate regulatory mechanisms that tumor cells can more rapidly achieve distant clonal growth.

In order to achieve long-distance metastasis, DTCs act as seeds that preferentially colonize specific organ niches characterized by favorable nutritional environments. The transcriptional and metabolic heterogeneity of DTCs facilitates the selection of clones capable of thriving in particular organs, while plasticity mechanisms dynamically induce adaptation to new microenvironments [111]. Clinically detectable metastases arise from successful metastasis-initiating cells (MICs) that have adapted to and selected their TME, ultimately achieving growth and organ colonization through the activation of regeneration, angiogenesis, and immunosuppressive programs [112]. Metastasis represents a continuous process involving reprogramming and clonal selection within varying microenvironments, alongside cancer cell subpopulations responding to selective environmental stressors. This dynamic leads to unchecked tumor proliferation, organ dysfunction, and systemic tissue impairment, and ultimately results in mortality.

## 6. Regulation of Microenvironment

The tumor microenvironment is a crucial factor in the induction of EMT and the initiation of metastasis. Mesenchymal stem-like cells within tumor niches interact with both tumor and stromal cells, facilitating processes such as vascularization, immune modulation, and extracellular matrix remodeling [113]. In this local microenvironment, hypoxia can significantly promote metastatic events. In tumor tissues hypoxiais, a significant feature of the microenvironment of solid tumors, mainly caused by multiple mechanisms, including abnormal blood vessels, high metabolic demands, limited oxygen diffusion, and changes in hemorheology. These mechanisms interact with each other, forming a complex vicious cycle that promotes tumor progression and treatment resistance. HIF-1α serves as an essential sensor and regulator of cellular oxygen levels; it protects cells from apoptosis under hypoxic conditions, enhances glycolysis, and diminishes mitochondrial OXPHOS in cancer cells [114].

Endothelial cells also play a pivotal role in cancer dissemination through their angiogenic functions. The integrity of endothelial cell–cell adhesion is maintained by VE-cadherin; however, its phosphorylation via RAC signaling pathways reduces cell–cell adhesion while increasing vascular permeability [115]. The mitochondrial protein Opa1 facilitates mitochondrial fusion, stabilizes mitochondrial calcium uptake 1 (MICU1) expression, enhances calcium ion uptake into mitochondria from the cytoplasm, inhibits the NF-kB pathway, influences angiogenic gene expression, and regulates both tumor angiogenesis and lymphatic vessel formation [116]. Conversely, when angiogenic factors such as VEGF are upregulated alongside hypoxia or energy restriction, leading to increased peroxisome proliferator-activated receptor gamma coactivator 1-alpha (PPARGC1A or PGC1α) expression, there is an acceleration of mitochondrial function that ultimately protects cellular viability [117]. Moreover, mitochondrial fusion not only provides energy but also participates in regulating complex cellular processes, including apoptosis, immunity, and cell differentiation. Currently identified oncogenes—such as c-Myc, Oct4, and K-Ras—and key cellular sensors like mTOR, AMPK, and HIF-1α are implicated in supporting metabolic adaptation during primary tumor growth [118].

In order to initiate migration, cancer cells depend on their metabolic plasticity to adapt energy production in response to environmental changes [119]. Depending on substrate availability, cancer cells reshape energy production pathways and regulate metabolic transitions between glycolysis and OXPHOS, exhibit preferences for mitochondrial oxidizable substrates, and synthesize intermediates of the TCA cycle [120]. To achieve metastasis, cancer cells must undergo metabolic adaptations that facilitate detachment from the ECM, local migration and invasion, as well as intravascular and extravascular infiltration into the bloodstream. Resistance to anoikis induced by detachment and overcoming growth signals received through attachment to the ECM are critical markers of the initial stages of metastasis.

Under conditions of metabolic stress, AMPK enhances pyruvate dehydrogenase (PDH) activity, catalyzing the conversion of pyruvate into acetyl–CoA. This process maintains substrate influx for the TCA cycle while supporting a metastatic phenotype [121]. After escaping from the primary tumor mass and entering circulation, CTCs reconnect their metabolism to survive by regulating mitochondrial ROS clearance. Invasive cancer cells utilize PGC1α, a transcriptional coactivator that promotes mitochondrial biogenesis and OXPHOS—an essential event in certain types of cancer related to movement and metastasis [122]. However, PGC1α exhibits variable contributions to cell invasiveness across different tumor types; for instance, it has been shown to reduce invasiveness in prostate cancer and melanoma models [123,124].

In the process of metastasis, the hallmark of ECM remodeling is characterized by increased deposition, fiber arrangement, and cross-linking, which collectively alter the rigidity of the TME. Integrin signaling has been shown to promote active cancer progression and metastasis. Certain solid tumors exhibit heightened ECM stiffness along with densely arranged collagen fibers that facilitate the escape of migrating cells from the primary tumor [125]. Within this microenvironment, metastatic cells modulate their metabolism to meet energy demands. A rigid ECM enhances mitochondrial fusion through activation of the β1-integrin/kindlin-2 signaling pathway [126]. Conversely, a softer ECM induces upregulation of DRP1 expression and promotes mitochondrial fission, thereby reducing cancer cell dissemination. Consequently, these findings suggest that novel anti-cancer strategies could target extracellular signaling pathways involved in metabolic communication to counteract metabolic adaptations driven by ECM mechanisms in solid tumors.

ROS, as byproducts of ETC activity, can activate HIF signal transduction pathways. Excessive ROS production may also lead to cellular apoptosis. Furthermore, extracellular metabolites present within the local niche significantly influence metastatic outgrowth. The niche-adaptive plasticity exhibited by cancer cells—coupled with innovative paracrine signaling—can induce similar plasticity in surrounding cells within target organs. This interaction fosters co-evolution between tumors and their new environments while facilitating the formation of a metastatic TME [127]. The initial metastatic niche can drive cellular reprogramming, which enables extensive secondary metastasis development, ultimately resulting in increased clinical incidence rates and mortality associated with these malignancies.

## 7. Emerging Perspectives on Therapy

Apoptosis is a programmed cell suicide process, characterized by mitochondrial fragmentation and dysfunction, nuclear condensation and other significant apoptotic markers of cell death. Research has shown that characterized apoptotic cancer cells can recover their normal morphology and proliferate after the removal of apoptosis inducers [128]. Anastasis is a natural cellular recovery pathway that enables cells to recover after removing the apoptosis-inducing agent [129]. This thus reveals a potential mechanism by which cancer cells might accidentally escape from chemotherapy. Anastasis can facilitate the survival of damaged or tumor cells, promote malignancy, and increase drug resistance and metastasis. Metastasis for cancer cells endowed with dynamic reprogramming capabilities, enabling them to adapt to various stresses, evade immune surveillance, and disrupt host tissue biology in order to facilitate tumor regeneration. This process is a value contributor to patient mortality. Furthermore, the same adaptive characteristics that confer stress resistance and promote tumor regeneration can be exploited by cancer cells to withstand treatment and regenerate tumors post-therapy. The intrinsic link between metastasis and resistance is evident; therapeutic interventions impose additional selection pressures on metastatic cancer cells, thereby fostering the emergence of tumor subclones harboring drug-resistance mutations while also triggering inflammatory signals that drive plasticity [130]. Notably, mitochondria are likely to play a pivotal role in these processes.

Metformin inhibits the mitochondrial respiratory chain complex and affects Ras-driven cancer stem cells [131]. Following the downregulation of Ras signaling genes, these cells exhibit an increased potential for synergistic effects when combined with oncogene-targeted therapies and metformin. The promotion of mitochondrial ROS generation to induce cancer cell death may enhance the efficacy of chemotherapy [132]. Consequently, targeting this ROS generation pathway could represent a promising anti-tumor strategy. Furthermore, certain drugs disrupt mitochondrial metabolic pathways, thereby preventing tumor cells from acquiring essential energy sources and metabolic intermediates. This interference with mitochondrial metabolism can inhibit autophagy and mitophagy, ultimately compromising mitochondrial function by obstructing quality-control mechanisms and/or substrate supply. Tumors exhibiting impaired mitochondrial function are compelled to upregulate glycolysis in order to satisfy their energy requirements [133].

Mitochondria play a pivotal role in the metabolic reprogramming of tumors and are closely associated with cancer treatment. The maintenance of mitochondrial function is critically dependent on the integrity of the mitochondrial membrane potential. Mitochondrial outer membrane permeabilization (MOMP) and the release of holocytochrome c are essential prerequisites for the development of resistance phenotypes [33]. Drugs that target mitochondrial membrane potential induce tumor cell death by disrupting this potential and causing mitochondrial dysfunction. Drug-tolerant persister cells, which evade apoptosis and resist conventional cancer therapies, represent a significant non-genetic barrier to effective cancer treatment [134]. ATF4 mitigates cellular stress and promotes survival by enhancing nutrient uptake, facilitating autophagy, and suppressing oxidative stress [135]. ATF4 mRNA translation initiates various downstream pathways that are essential for the persister phenotype, including metabolic reprogramming, cell cycle inhibition, immune evasion, and EMT induction. Consequently, pharmacological agents targeting ATF4 or associated pathways may exacerbate mitochondrial dysfunction in tumor cells by inhibiting mitophagy and subsequently induce cell death.

## 8. Conclusions

Metastasis is a complex and uncertain pathological event that arises from the interplay between metastatic cells and various microenvironmental factors. Rather than entering a dormant state, cancer cells can exploit the pre-metastatic niche; thus, it is essential to elucidate the intricate intercellular signaling involved in communication processes among primary cells, quiescent cells, and metastatic cells, as well as their interactions with matrix components and cell state trajectories. Mitochondria play crucial roles in the signal transduction, proliferation, apoptosis regulation, invasion, and migration of cancer cells. Firstly, by specifically targeting and eliminating the mitochondria of tumor cells—particularly through the inhibition of mitochondrial RNA polymerase and other methodologies—it is possible to induce mitochondrial autophagy by targeting mitochondrial membrane proteins, triggering inflammatory responses, and mediating cell death. Secondly, immune cells can acquire information regarding mitochondria mutations in tumor cells, thereby adjusting their thresholds at immune checkpoints. The exchange of information between mitochondria and the endoplasmic reticulum, along with mitochondrial proteins, has the potential to enhance the vitality of immune cells and bolster their anti-tumor capabilities. Importantly, mitochondria have emerged as a pivotal drug target for preventing metastasis and tumor treatment in the future.

## Abbreviations

ATP, adenosine triphosphate; OXPHOS, oxidative phosphorylation; ROS, reactive oxygen species; mtDNA, mitochondrial DNA; nDNA, nuclear DNA; TME, tumor microenvironment; UPR, unfolded protein response; ER, endoplasmic reticulum; MAM, mitochondria-associated ER membrane; OMM, outer mitochondrial membrane; TCA, tricarboxylic acid; FAO, fatty acid oxidation; AMPK, AMP-activated protein kinase; NO, nitric oxide; EMT, Epithelial–mesenchymal transition; EMT-TFs, EMT transcription factors; mTORC1, mechanistic target of rapamycin complex 1; NRFs, nuclear respiratory factors; PGC-1, PPAR-γ coactivator 1; FAs, focal adhesions; MICs, Metastasis-initiating cells; MMP, mitochondrial membrane potential; CTCs, circulating tumor cells; ATF4, activating transcription factor 4; CKAP4, cytoskeleton associated protein 4; REDD1, Regulated in Development and DNA Damage Response-1; HIF-1, hypoxia-inducible factor 1; IFRD1, Interferon-related developmental regulator 1; TRP53, p53 tumor suppressor gene; DTCs, disseminated tumor cells; GLS1, Glutaminase 1; CSC, cancer stem cell; OCT4, organic cation/carnitine transporter4; SOX2, SRY-box transcription factor 2; KLF4, KLF transcription factor 4; c-MYC, myelocytomatosis oncogene; iPSCs, induced pluripotent stem cells; mPTP, mitochondrial permeability transition pore; MET, Mesenchymal–epithelial transition; TGN, trans-Golgi network; Arf1, ADP ribosylation factor 1; mtF-actin, mitochondrial actin filaments; MICU1, mitochondrial calcium uptake 1; MOMP, Mitochondrial outer membrane permeabilization.
